# Policies and practices in Nigeria’s pharmaceutical sector: A mixed methods exploration of stakeholders’ perspectives on strategic reforms

**DOI:** 10.1016/j.hpopen.2023.100091

**Published:** 2023-03-24

**Authors:** Obi Peter Adigwe

**Affiliations:** National Institute for Pharmaceutical Research and Development, Plot 942, Cadastral Zone C16, Idu Industrial District, Abuja, Federal Capital Territory, Nigeria

**Keywords:** Pharmacy practice, Stakeholders, Policy, Development, Medicines’ Security

## Abstract

**Background:**

Policies and practices are key factors that determine development in any sector. In the Nigerian setting however, there is lack of evidence that the pharmaceutical sector is embedded with contextual policies and practices that can expedite development in the system. This inadvertently has an impact as regards access to medicines for the citizenry. This study therefore aimed at adopting a bottom-up approach in gathering insights into stakeholders’ perspectives on policies and practices in Nigeria’s pharmaceutical sector, and how they influence Medicines’ Security and consequent access to healthcare.

**Methods:**

Data were collected using a self completion questionnaire which was administered to stakeholders present during an event held in Abuja, the capital of Nigeria, which focused on improving the Nigerian pharmaceutical sector. A total number of 82 questionnaires were administered to participants. Following retrieval of questionnaires, quantitative data were subjected to descriptive and inferential analyses, whilst textual data were analysed using thematic analysis approach.

**Results:**

Of the 82 questionnaires administered, response rate was 92.68%. Two-thirds of the participants were males (69.7%). A quarter of the study participants were between the ages of 41 and 50 years, whilst those above 50 years represented the most populous proportion of the sample (38.2%). A considerable proportion (48%) of the study participants indicated that current policy ecosystem was hostile for pharmaceutical sector growth and development. Also, majority (97.3%) of the study participants indicated that increased investment in health research could stimulate the development of the pharmaceutical sector. Majority of the study participants indicated the need for collaboration between pharmaceutical companies, research institutes and the petrochemical industry.

**Conclusion:**

This study consequently identified several critical factors that could stimulate development in the sector, including increased funding of research; stringent implementation of existing policies; and prioritisation of pharmaceutical sector by government and other key stakeholders.

## 1 Background

The Nigerian pharmaceutical sector is a multibillion dollar industry with underutilised potential that can contribute to the economy as well as improve the quality of lives of its citizens. The pharmaceutical industry is therefore considered a critical component of the manufacturing sector and has been reported as an important contributor to the Nation’s Gross Domestic Product (GDP) [1]. About a decade ago, a seminar report enumerated 130 local drug manufacturers in Nigeria and identified that this represented about a third of the total pharmaceutical manufacturing capacity in the West Africa sub region [2]. Despite this apparent capacity, local manufacturing has not been perceived as making commensurate impact on medicines’ access in Nigeria [3]. Experts therefore identified that developing local pharmaceutical manufacturing capacity as a means of strengthening the sector, will enable it harness local raw materials and natural flora which are abundant in the country [4]. This approach has been identified as one of the most sustainable pathways towards the development of new drugs as well as a means of improving the availability of existing formulations [5]. Additionally, the approach is also seen as a critical tool for the achievement of National Medicines’ Security, by reducing the inordinate dependence on importation of pharmaceutical products, especially those which local companies have the capacity to manufacture [4].

There is evidence that suggests that given the considerable manufacturing capacity in Nigeria, local production can satisfy up to three quarters of national medicines’ needs [6]. Paradoxically, capacity utilisation of the industry is below 30% and about 70% of medicines consumed in Nigeria are imported into the country [7]. The current status of the Nigerian healthcare sector where majority of its medicines are imported, is in direct contravention of the National Drug Policy. The policy was formulated in 1990, and when it was revised in 2005, it was designed to ensure that within a decade of revision, majority (70%) of national medicines’ needs would be satisfied through local manufacturing. In line with the achievement of this objective, the National Drug Policy also sought to promote pharmaceutical research and development of raw materials for manufacturing pharmaceutical and related products. The policy further aimed at increasing availability of high quality, effective, affordable, and safe medicines, through various relevant means [8], [9], [10].

The Medicines’ Security concept is critical not only to how nations plan citizens’ access to high quality, safe, and efficacious medicines, but also to the development of sustainable systems that address contextual socioeconomic needs such as unemployment, knowledge deficits and revenue generation [11]. Given that about 10 million people worldwide die every year due to poor access to essential medicines [12], and that Nigerians are disproportionately affected [13], understanding the interactions between pharmaceutical sector policies and practices, as well as their influence on access to healthcare is not only critical, but also expedient. So far, very few experts have sought to address this knowledge gap. A decade-old study revealed that out of the 130 pharmaceutical manufacturing companies in Nigeria, less than half were in active operation [14]. This highlights the urgent need for contemporary evidence to underpin policy and practice reforms.

A better understanding of current policies and practices in Nigeria’s pharmaceutical sector is invaluable in developing contextual strategy that can address sectoral deficiencies. This is especially important, considering the fact that previous policies have not been able to achieve the required developmental objectives [8], [9], [10]. A review of the extant literature revealed that no study had undertaken a robust and comprehensive interrogation of critical issues in the context of contemporary challenges facing both the sector and the nation. This study therefore aimed at adopting a bottom-up approach in gathering insights into stakeholders’ perspectives on policies and practices in Nigeria’s pharmaceutical sector, and how they influence Medicines’ Security and consequent access to healthcare.

## 2 Methods

### 2.1 Study design

A cross sectional study was undertaken in the Federal Capital Territory, Nigeria using self administered questionnaire. The study participants were stakeholders present at an event targeted at Nigerian pharmaceutical sector development. The stakeholders include pharmaceutical industry practitioners, healthcare professionals, researchers, policymakers and development partners.

### 2.2 Study tool

Following an extensive literature review [1], [2], [3], [8], [12], a questionnaire was designed to assess views of respondents on extant policies and practices in Nigeria’s pharmaceutical sector, as well as on strategies to improve the system. The questionnaire was made up of items that explored socio-demographic characteristics and items relating to relevant pharmaceutical sector development factors. A Likert scale of 1 to 5 was employed for some of the questions and they were represented as follows: 1 = Strongly Disagree, 2 = Disagree, 3 = Neutral, 4 = Agree, and 5 = Strongly Agree. Other relevant open-ended items were included to enable the collection of relevant qualitative data. The questions indicated in the qualitative section include views about strategies to develop the pharmaceutical sector and views about the potential in the Nigerian pharmaceutical sector.

### 2.3 Questionnaire validation

Face and content validations of the data collection instrument were undertaken using a team of experts comprising 3 persons who were involved in teaching and research activities in this field. The questionnaire was assessed for appropriateness, complexity, and attractiveness. Content validity ratio and content validity index tests were undertaken for each item, and only items that passed these tests were included in the final questionnaire. A pilot testing of the questionnaire was carried out by administering it to 15 randomly selected participants who are practitioners in the pharmaceutical sector. The feedback received did not necessitate any major change.

### 2.4 Ethical consideration

Prior to data collection, ethical clearance was granted by the National Institute for Pharmaceutical Research and Development Health Research Ethics Committee with approval number HREC 11/04/2020–05. Ethical principles of confidentiality and anonymity were maintained throughout the data collection process. Some measures adopted to achieve the aforementioned, include the exclusion of names and other identifying features from the data collection tool. Participation in the study was voluntary, as informed consent was obtained from the participants prior to questionnaire administration.

### 2.5 Data collection

The validated study tool was administered to stakeholders during the event. Questionnaires were completed and returned after completion. A convenience sampling strategy was employed [15], and every-one present at the conference was approached to complete the questionnaire. Inclusion criteria were; participants present at the conference, willingness and consent to participate. Participants who did not meet the criteria above were excluded from the study. A total of 82 questionnaires were administered, and the purpose of the study was explained to the participants before administration.

### 2.6 Data analysis

Following retrieval of questionnaires, quantitative data were coded directly into Statistical Package for Social Sciences (SPSS) version 21, and verified for consistency, correctness, and completeness before analysis [16]. Descriptive statistics was carried out, and association of responses with socio-demographic characteristics was determined using chi square test. A *p value* of 0.05 or less represented the threshold for statistical significance. Qualitative data collected from the open ended items on the questionnaire were subjected to thematic analysis [17].

## 3 Results

### 3.1 Demography

A total number of 76 questionnaires were completed and returned, giving a response rate of 92.68%. Female participants were in the minority as indicated by 30.3% of the sample. A quarter of the study participants were between the ages of 41 and 50 years, whilst those above 50 years represented the most populous proportion of the sample (38.2%). Occupationally, public and civil servants represented more than half of the study participants surveyed (63.2%), whilst politicians and policymakers represented the least proportion (2.6%). Further details of the cohort's socio-demographic characteristics are presented in Table 1.

**Table 1:** Socio-demographic characteristics.

**Variables** **n = 76**	**N (%)**
**Gender**	
Male	53 (69.7)
Female	23 (30.3)
**Age**	
18–30	9 (11.8)
31–40	16 (21.1)
41–50	19 (25.0)
51 and Above	29 (38.2)
Missing Data	3 (3.9)
**Highest Education Level Achieved**	
Diploma (OND)	1 (1.3)
First Degree (or HND)	29 (38.2)
Master’s Degree	29 (38.2)
Doctorate Degree	16 (21.1)
Missing Data	1 (1.3)
**Occupation**	
Public/Civil servant	48 (63.2)
Business person	10 (13.2)
Politician or Policymaker	2 (2.6)
Development/International Agency	6 (7.9)
Others	7 (9.2)
Missing Data	3 (3.9)

Note: HND = Higher National Diploma, OND = Ordinary National Diploma.

### 3.2 Quantitative findings

#### 3.2.1 Developing a robust and comprehensive strategy

**Fig. 1:**
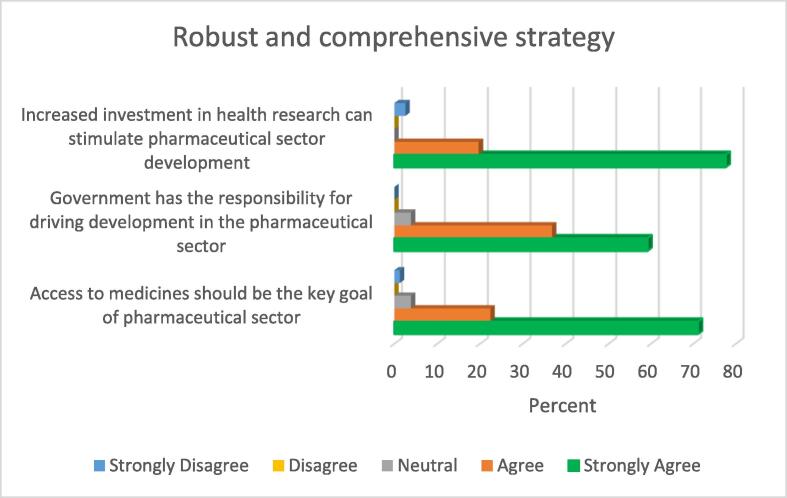
Robust and comprehensive strategy.

The healthcare system depends on medicines to function effectively, and the pharmaceutical sector is responsible for production and distribution of medicines necessary to achieve optimum results. From Fig. 1, it can be seen that 93.5% of the study participants indicated that access to medicines should be the key goal of pharmaceutical sector development. Almost all (96%) of the study participants agreed that the responsibility for initiating and driving development in the sector lies with the Government.

Majority of the study participants indicated that increased investment in health research could stimulate the development of the pharmaceutical sector as 97.3% of the participants indicated “strongly agree” and “agree” in response to this item.

#### 3.2.2 Actualising potentials of Nigerian pharmaceutical sector

**Fig. 2:**
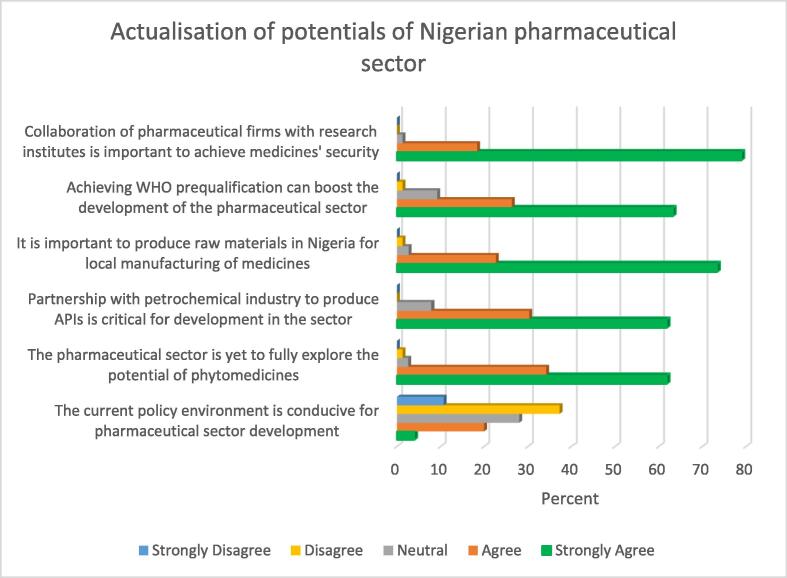
Potentials of Nigerian pharmaceutical sector.

A favourable policy environment is an important factor for the growth of any sector. In this study, close to half (48%) of the study participants indicated that the current policy environment was not conducive for the growth of Nigerian pharmaceutical sector. Almost all (96%) the study participants however indicated that the potentials of phytomedicines and natural resources had not been fully explored. Majority (92.1%) of the study participants indicated that there was a need to partner with petrochemical industry to produce Active Pharmaceutical Ingredients (APIs). Other relevent details are provided in Fig. 2.

From Fig. 2, almost all (96%) the study participants indicated that it was important to produce raw materials for the production of medicines in Nigeria. Majority (89.5%) of the study participants indicated the need for Nigerian organisations to attain WHO prequalification, as this can boost the development of Nigerian pharmaceutical sector. Almost all (97.3%) the participants indicated that pharmaceutical manufacturing firms should collaborate with research institutes so as to enhance their productivity.

#### 3.2.3 Interests, perceptions and insights

The results presented in this section are multiple responses. From the findings, majority of the study participants were more interested in research and academics (75%), compared to other aspects of the sector. On the contrary, the practice area which emerged as the least interesting to the study cohort was importation (13.60%). It is however noteworthy that non-traditional areas such as policymaking (42.11%), public health (40.79%) as well as supply chain and logistics (35.22%) emerged as areas of significant interest for the study cohort. Other areas of interest indicated by the participants include manufacturing (28.95%), hospital practice (35.52%), community practice (22.37%), and traditional medicine (23.68%).

#### 3.2.4 Association between socio-demographic characteristics and some variables

Further to the descriptive statistical analysis undertaken on the data, cross tabulation was also undertaken and chi square test was used to determine association between socio-demographic characteristics and some of the variables. Findings revealed that some of the responses of the study participants were influenced by their socio-demographic characteristics, as chi square test was significant for some of the items. A strong majority of public servants agreed that access to medicines should be the key goal of pharmaceutical sector development, compared to only half of policymakers that agreed with the same concept. This finding was statistically significant (*p* = 0.007).

Another interesting finding that emerged at this level of analyses, was in relation to respondents’ perceptions on strategies for actualising the potentials of the Nigerian pharmaceutical sector. The findings revealed that participants’ views differed significantly, based on their level of education. There was a strong agreement amongst degree holders and postgraduate respondents on the need for pharmaceutical manufacturing firms to collaborate with research institutes to enhance their productivity. On the other hand however, respondents who were not educated up to degree level disagreed with this strategy. This finding too was statistically significant (*p* = 0.0001). Further details are provided in Table 2.

**Table 2:** Association between socio-demographic characteristics and variables.

**Variables**	**Demography**	**Strongly Disagree (%)**	**Disagree (%)**	**Neutral (%)**	**Agree (%)**	**Strongly Agree (%)**	**X^2^**	***p-*value**
Access to medicines should be the key goal of Pharmaceutical Sector development	Public/ Civil Servant	–	–	4.2	29.2	66.7	27.189	0.007
	Businessperson	10.0	–	–	–	90.0		
	Politician or Policymaker	50.0	–	–	–	50.0		
	Development/ International Agency	–	–	–	16.7	83.3		
	Other	–	–	14.3	28.6	57.1		
There is need for the pharmaceutical manufacturing firms to collaborate with research institute to enhance their productivity	Diploma (OND)	–	100.0	–	–	–	77.436	0.0001
	First Degree or (HND)	–	–	–	17.2	82.8		
	Master’s Degree	–	–	–	28.6	71.4		
	Doctorate Degree	–	–	–	6.3	93.8		

### 3.3 Quantitative findings

Textual data provided by the study participants in relation to the development of the pharmaceutical sector were analysed thematically following the qualitative research paradigm. Four themes emerged from the qualitative analysis.

#### 3.3.1 Collaboration amongst stakeholders

In relation to the development of the pharmaceutical sector, it emerged that the adoption of a collaborative approach involving all relevant stakeholders, was perceived as most appropriate for the Nigerian setting.

*“…all stakeholders have to be involved to achieve a robust and comprehensive development” (Male Participant, Public Servant, above 51 Years)*.

*“…continuous collaboration with other stakeholders would help improve the current situation” (Male Participant, Pharmacist, above 51 Years).*

#### 3.3.2 Responsibilities of government

The study respondents further identified government as the key stakeholder responsible for articulating, driving and organising the relevant reforms in the sector.

*“Government needs to demonstrate commitment to pharmaceutical sector as a national priority” (Male Participant, Public Servant, above 51 Years).*

*“…there must be a government structure to ensure policy implementation” (Male Participant, Development Sector Professional, 41 – 50 Years).*

*“Government should not take sole responsibility on investing but private companies, international organisations should partake and partner in drug production” (Female Participant, Public Servant, 18 – 30 Years).*

In addition to outlining government’s role and responsibilities, the findings from the qualitative part of the study further corroborated findings from the quantitative aspect, particularly with respect to identifying the need for partnership with the private sector and with development partners.

#### 3.3.3 Adapting new technologies

From the study participants’ perspective, there was a need for the Nigerian Pharmaceutical sector to embrace emergent new technologies in various aspects of the sector as a catalyst for the overarching development of the sector.

*“Adaptive technology should be put in place properly to improve the development of the Pharmaceutical Sector” (Female Participant, Public Servant, 18 – 30 Years).*

*“…many researches [sic] conducted are not transformed into products” (Male Participant, Public Servant, above 51 Years).*

In addition to the need to integrate new technologies in the Nigerian setting, participants also identified a lack of a framework that supports translational research, resulting in a situation where most research output are left on the shelf.

#### 3.3.4 Unexplored socioeconomic potentials

Another important theme that emerged in the qualitative aspect of this study was the hitherto unexplored socioeconomic potentials of the pharmaceutical sector

*“Leveraging on local research outcomes should be considered by pharmaceutical industries to support local production” (Male Participant, Public Servant, 31 – 40 Years).*

*“The potential of Nigeria pharma sector is large and if tapped, it will increase the economic indices of Nigeria” (Female Participant, Public Servant, Above 51 Years).*

The findings from the thematic analysis have identified strong linkages between local manufacturing and national economic growth. These findings which are at the very core of the Medicines' Security concept advocate the strengthening of local pharma manufacturing through contextual research, as a critical tool for improving access to healthcare. Concurently, the achievement of socioeconomic objectives such as revenue generation, job creation and capacity building, are also priortised.

## 4 Discussion

This study has provided novel insights regarding contextual policies and practices in Nigeria’s pharmaceutical sector. A strong majority of the participants indicated that access to medicines should be the key goal of the pharmaceutical sector whilst they also opined that government has the responsibility for initiating development in the sector. Investment in health research was considered a sustainable strategy for stimulating relevant development in the sector. Participants identified the current policy environment as unfavourable for growth, and indicated the need for policy reforms. The thematic analysis further provided evidence that deepened the socioeconomic aspects of the Medicines’ Security concept. The correlations between local manufacturing underpinned by contextual research and stimulation of critical socioeconomic indices, represents novel findings in both the subject area the study setting.

Over the years, the issue of access to affordable essential medicines in healthcare systems has been a matter of global concern [18]. Studies have revealed that the health of a country’s general population is significantly affected by access to healthcare and availability of medicines [12], [19]. It is therefore important for policymakers, practitioners and other stakeholders in the sector to continuously explore novel mechanisms to enable the achievement of Medicines’ Security. What this study adds, is the identification of development partners and the corporate sector as key stakeholders, plus the need for collaborative working in policy reforms especially articulation, development and implementation.

In this study, study participants indicated the need for government to increase investment in health research as a way of stimulating the development of the sector. A similar finding was previously reported by Sun *et al.* [20] in China. The findings from the study in this thematic area is in tandem with the pressing need to build capacity in health research, particularly in Nigeria’s developing healthcare setting, where the gap is seemingly daunting. For policymakers, it is important to look at funding holistically, with a view to exploring alternative sources for funding health research. Also, it is important to fully implement existing policies which have prospects of developing Nigeria's pharmaceutical sector. There are existing policies that can catalyse the development of the pharmaceutical sector, if well implemented. An example of such policies is the National Drug Policy [9]. The National Drug Policy set a target of 70% for local manufacturing of medicines consumed in Nigeria, however there is little evidence that this target has been achieved, after more than a decade of implementation. This perhaps emphasises study participants’ exhortation of the improved collaboration and active participation of critical stakeholders in the articulation, development and implementation of pharmaceutical sector policies. Although this study represents the first time a collaborative, multi-stakeholder participation is being promoted for the entire policy ecosystem for the sector, aspects of this strategy have been identified as effective [21].

Findings from this study identified current policies as hostile for pharmaceutical sector development, suggesting desperate and urgent reforms to stimulate necessary reforms. Despite the challenges faced by the sector, majority of the study participants were of the view that the Nigerian pharmaceutical sector possesses significant potentials especially in the area of phytomedicines and natural resources. These are resources, which when properly harnessed can stimulate the production of APIs, excipients and other pharmaceutical raw materials. This finding is in line with other previous studies that indicated the need to focus on exploring medicinal plants and other natural resources, as a means of improving access to healthcare services [22], [23]. The fact that majority of the study participants indicated agreement with the stated item is a strong indication of a good understanding of the inherent potential of phytomedicines for both the pharmaceutical industry and the healthcare sector. A significant proportion of all medicines were derived from plants and today, many pharmaceutical classes of drugs include a natural prototype [24]. Traditionally, herbs and phytomedicines have provided very useful synthetic clues for orthodox medicines [25]. Medicinal plants have also been recognised as an unparalleled source of molecular diversity for drug discovery and development [26]. Whilst this work provides evidence that validates advantages associated with phytomedicines, the heightened interest in research revealed in the study represents an opportunity to develop contextual strategies that can appropriately harness these opportunities.

Participants in this study indicated that manufacturers need to partner with the petrochemical industry to locally produce APIs and other raw materials. Implementing this recommendation can help reduce inordinate importation of pharma input; build capacity of industry actors; and create employment in various relevant settings [27]. Also, local production of APIs will significantly contribute to the assurance of Medicines’ Security and consequently improve access to healthcare in the country. Partnership between the petrochemical sector and pharmaceutical sector stakeholders will also improve efficiencies in the achievement of Government’s overarching objectives, especially with respect to diversification of revenue streams. With respect to WHO prequalification, there was a strong consensus amongst the study participants on the need for local pharmaceutical companies in Nigeria to consider this quality improvement approach. Presently, no local pharmaceutical firm in Nigeria is on the WHO prequalified list [28]. The achievement of WHO prequalification by local manufacturers can enhance relevant development in the sector, as well as improve potentials for export.

Regarding collaboration, findings from this study indicate that partnership between local pharmaceutical manufacturers and research institutes is necessary for achieving Medicines’ Security, these findings are in agreement with a previous study [29]. Available evidence suggests that collaboration between pharmaceutical companies and research institutes was poor, and this has impacted the pharmaceutical sector negatively [1]. It is therefore urgent and important to initiate policy reforms that will promote collaboration between research institutes and local pharmaceutical companies. Successfully achieving this will expedite the translation of research activities into products and processes. This is particularly important at this point in time, given the increasing interest in research as indicated by the emergent findings of this study.

## 5 Limitation and strength

Possible limitations of this study are associated with the convenience sampling strategy adopted and the small size of the sample. Despite these potential weaknesses, this study adopted a novel mixed methods approach which provided new insight into critical policies and practices expected to drive development in Nigeria’s pharmaceutical sector, especially from stakeholders’ perspectives. Further research is however required to deepen some of these emergent findings, as well as to determine specific barriers preventing the implementation of existing policies which have the potentials to catalyze development of Nigerian pharmaceutical sector.

## 6 Conclusion

This exploratory study adopted a novel mixed methods approach to interrogate stakeholders' perceptions of pharma sector policies and practices. Increased collaboration between pharmaceutical companies, research entities, and the petrochemical industry was identified as critical for the production of APIs, excipients, phytomedicines and other inputs. Consequently, the sector must begin to look inwards as regards harnessing local resources available within the country for the development of contextual interventions and solutions.

In the study setting, the prioritisation of contextual pharmaceutical sector research and the adoption of new technologies emerged as catalysts for improving access to medicines and enabling achievement of critical socioeconomic indices. The findings of this study can prompt the development of contextual and responsive initiatives that address vulnerabilities in the pharmaceutical sector. The resulting reforms of industry policies and practicies of the aforementioned, can consequently contribute to the achievement of Medicines’ Security and Universal Health Coverage in Nigeria..

## Declaration of Competing Interest

The authors declare that they have no known competing financial interests or personal relationships that could have appeared to influence the work reported in this paper.
